# Influence of Microstructure and Shot Peening Treatment on Corrosion Resistance of AISI F55-UNS S32760 Super Duplex Stainless Steel

**DOI:** 10.3390/ma11061038

**Published:** 2018-06-19

**Authors:** Andrea Francesco Ciuffini, Silvia Barella, Luis Borja Peral Martínez, Carlo Mapelli, Inés Fernández Pariente

**Affiliations:** 1 Dipartimento di Meccanica, Politecnico di Milano, via La Masa 34, 20156 Milano, Italy; silvia.barella@polimi.it (S.B.); carlo.mapelli@polimi.it (C.M.); 2 Escuela Politécnica de Ingeniería de Gijón, Universidad de Oviedo, Campus de Viesques, Gijón 33203, Spain; uo195967@uniovi.es (L.B.P.M.); inesfp@uniovi.es (I.F.P.)

**Keywords:** super duplex stainless steels, shot peening, microstructure, x-ray diffraction, salt spray fog

## Abstract

Shot peening is a surface process commonly used in the aeronautic and automotive industries to improve fatigue resistance. Shot peening is proven to be beneficial in the fatigue behavior of components, but rarely has its influence on wear and pitting corrosion resistance been evaluated. In this work, shot peening was performed on AISI F55-UNS S32760 super-duplex stainless steel samples previously submitted to various thermal treatments, to obtain different initial microstructures and properties. Samples have been characterized in terms of microstructure morphology, local chemical composition, microhardness of each constituent phase, and energy dissipation modes. The enhanced properties provided by shot peening has been evaluated through residual stress depth profiles and Full Width at Half Maximum (FWHM) using X-ray diffraction (XRD), surface hardness, surface roughness, and corrosion resistance through salt spray fog tests. The 1400 °C solution thermal treatment was identified as the optimum initial condition, which maximizes the advantages of the shot peening treatment, even pitting corrosion resistance. These results are related to the uniformity of austenite and ferrite in terms of microstructure morphology, micromechanical properties, and alloying elements distribution.

## 1. Introduction

Nowadays, with the intent to improve fatigue strength and to increase the life span of components, several processes are employed. Shot peening is one of the most widespread processes that can improve fatigue behavior. It is widely used and is a low cost and simple method. Its application is still further expanding especially in aeronautic and automotive industries [1,2,3].

Shot peening consists of impacting a flux of spherical shots against a metallic surface, inducing a residual stress field and work hardening on the top layer of the components. Due to the shot impacts, the surface layer of a component yields plastically. The result from the inability of the plastically deformed material to rearrange itself on the elastic subsurface layers generates residual compressive stresses. At the same time, because of the shot marks the surface roughness increases and work hardening is induced a result of the plastic deformation. The beneficial effects of this mechanical surface treatment are mostly related to the compressive residual stress field and the induced strain hardening [4,5,6,7]. On the surface, residual compressive stresses and work hardening are able to stop difficult crack propagation, reducing the probability of fatigue damage. Thus, the increase in resistance to fatigue, stress corrosion cracking, fretting, galling, erosion, and closing pores are common benefits that result from the shot peening process [8,9,10,11,12,13,14,15,16].

Regarding stainless steels, in recent years it has been found that the corrosion potential becomes nobler with an increase in compressive stresses at the surface layer, since the Cr/Fe ratio in the passive film increases where large compressive stresses are found in the grains under it. Then, thanks to these compressive stresses, this passive surface layer can result in a higher corrosion resistance [17,18,19,20].

Thanks to their biphasic microstructure and high content of alloying elements, such as chromium and nickel, duplex and super duplex stainless steels feature good pitting corrosion resistance, which promotes their exploitation in oil & gas, chemical, and marine applications where high corrosion resistance is usually required. Thus, in this way, residual stress fields could have a beneficial effect, since they have been found to increase pitting corrosion resistance in stainless steels [17,18,19,20].

Bearing these ideas in mind, the aim of this work is to perform shot peening treatments to a F55-UNS S32760 super duplex stainless steel, in order to study the influence of this treatment on corrosion resistance.

In detail, the influence of various initial conditions of F55-UNS S32760 super duplex stainless steel specimens prepared through different thermal treatments are evaluated in this work. The investigated thermal paths avoid the precipitation of detrimental intermetallic phases, fulfilling the classical standard requirements. Nonetheless, the physical properties of the target material are strongly affected by its thermal history, both in terms of mechanical properties and microstructure morphology [21,22,23,24].

Shot peening treatments were performed after the thermal treatments of the samples. Characterization has been made in terms of microstructure morphology, local chemical composition, microhardness of each constituent phase, and energy dissipation modes. The enhanced properties that are a result of the shot peening have been evaluated in terms of residual stress depth profiles, FWHM (Full Width at Half Maximum, related with the work hardening), both by means of X-ray diffraction (XRD), surface hardness, surface roughness, and corrosion resistance through salt spray fog tests. The influence of the initial thermal treatments on the results have been highlighted.

The shot medium employed was Zirshot^®^ Y300, composed of ceramic beads and blasted with a 10A Almen intensity. Then, the microstructure, the surface hardness, the residual stresses field, and the corrosion resistance properties of the shot peened specimens have been evaluated and the differences, generated by the various initial condition of the target material, have been highlighted.

## 2. Experimental Procedure

### 2.1. Material, Specimens, and Thermal Treatments

Specimens have been drawn from a commercial AISI F55-UNS S32760 (Eure Inox S.r.l., Peschiera Borromeo, Italy) cold drawn bar, drawn at about 1 ms^−1^ in three passes with a section area reduction of approximately 25%. Their dimensions were approximately 25 × 25 × 10 mm^3^. A first set of samples was taken as a reference and named “CD” (from “Cold Drawn”) in the following. Afterward, thermal treatments were performed in different series of specimens, in order to modify their microstructure and properties. The first solution thermal treatment was performed at 1300 °C for 60 s per millimeter and then all the samples were water quenched. This thermal treatment was executed to achieve a duplex microstructure, composed only by α-ferrite and γ-austenite, to prevent the formation of an undesired secondary phase. Further, this thermal treatment removes the residual stresses and deformation patterns, derived from the previous forming process. Through this thermal treatment, a supersaturation of γ-former elements within the α-ferritic phase was also obtained, as depicted by the phase diagrams. The thermal and chemical homogenization was ensured by the 60 s/mm soaking time [25,26].

Subsequently, an annealing thermal treatment at 1080 °C was executed with different holding times: 36, 72, 210, and 360 s/mm. The corresponding samples were named in the following as: “A36”, “A72”, “A210”, and “A360”. This thermal treatment was performed with the aim of reproducing the temperature conditions that are currently employed during the real hot-working process, again, for the purpose of avoiding the precipitation of the detrimental and embrittling secondary phases. Then, all the specimens were water quenched.

Finally, other samples were annealed at 1300 °C and 1400 °C for 72 s/mm and water quenched, in order to also evaluate the effect of the thermal treatment temperature solubilization. These specimens were named as “S1300” and “S1400” [21].

The performed thermal treatments are summarized in Table 1.

The investigated microstructures were chosen due to their industrial interest. 1300 °C is a typical solution annealing temperature before forging. The 1080 °C annealing is the classical hot forming temperature and post-forming recrystallization/homogenization annealing. The microstructure that results after a solution thermal treatment at 1400 °C is the same one that results after fast solidification in welds. A second annealing after the 1400 °C solution treatment will lead to a microstructure featured by coarse Widmanstätten secondary austenite precipitates, which are well-known to be extremely deleterious for toughness. Due to this reason, these microstructures have not been investigated in this work [21].

### 2.2. Shot Peening Treatment

All samples have been polished with a 400-grit diamond abrasive (R_a_ around 12 µm), since the surface of the specimens after the thermal treatment were covered by scales that had to be removed. Further, this ensures that the samples have a uniform initial condition before shot peening.

Then, some specimens from each series were submitted to the shot peening treatment in a Guyson International Euroblast 4PF Blast Cabinet^®^ machine (Guyson International Ltd., Skipton, UK). Saint-Gobain ZirPro Zirshot^®^ Y300 ceramic beads (300 μm diameter and 700 HV, SEPR-Saint-Gobain ZirPro, Le Pontet CEDEX, France) were used as shots. Almen intensity was set at 10A, using an impact angle of 90°, and 98% coverage and defined as “full coverage”. The time necessary to achieve this level of coverage was obtained using the Avrami equation [27]. The choice of the Almen intensity was driven by previous studies where an increase in fatigue resistance for duplex stainless steels had been already achieved using similar treatment parameters but different blasting mediums [28,29].

Table 2 shows the data of the Avrami equation. The autoregressive (AR) parameter was obtained after 1 s of blasting exposure. The coverage after 1 s of SP was determined using image processing, five images were taken for each sample in different areas [27]. The results of the coverage after 1 s of SP and its uncertainty are reported hereafter in Table 2. Using these data to solve the Avrami equation, the exposure time necessary to achieve full-coverage has been calculated. An experimental aspect, which may have influenced the variation of the time to full-coverage, is the distance from the SP nozzle. Indeed, the initial dimensions of the samples were quite similar (approximately 25 × 25 × 10 mm^3^), but during the heat treatment they experienced a different hot-oxidation and, consequently, scale formation. Thereafter, all samples were polished with a 400-grit diamond abrasive (R_a_ around 12 µm), since the surface of the specimens after thermal treatment were covered by scales that had to be removed. Further, this ensures that the samples have a uniform initial condition before shot peening.

### 2.3. Optical Microscopy

Specimens have been prepared for optical microscopy using Beraha’s tint etch, following the standard ASTM E407-07 [30]. The solution composition is here reported: H_2_O 100 mL, HCl 20 mL, 0.6–1.0 g of K_2_S_2_O_5_ and 2–10 g (NH_4_F) HF. This chemical etching highlights the biphasic microstructure of the investigated alloy, differently coloring the α-ferrite and γ-austenite. Afterwards, following the ASTM E1245-13 [31] and ASTM E1382-10 [32] standards, the quantitative metallographic analysis was performed. The specimens were electrochemically etched at 10 V for 15 s with oxalic acid solution (10 g of H_2_C_2_O_4_ in 100 mL of H_2_O 100 mL). This etch allowed the observation of twins within the austenitic phase, which were counted following the ASTM E1245-13 and ASTM E1382-10 standards [31,32].

### 2.4. Austenite Volume Fraction Measurements via X-ray Diffraction

The optimization of the properties of the super duplex stainless steels was obtained with the proper control of the chemical composition and processing conditions, to obtain about an equal proportion of the austenite and ferrite [23]. For the sake of clarity, the microstructure was featured through the volume fraction of the austenitic phase, expressed as a percentage [33].

Austenite volume fraction of the samples was measured with a Stresstech Xstess 3000 G3R^®^ diffractometer (Stresstech Oy, Vaajakoski, Finland) to evaluate any possible percentage variation related to the shot peening process. These tests were performed using Kα-Chromium radiation coupled with a Vanadium filter and following the ASTM E975-13 standard [34].

### 2.5. Residual Stresses Measurements via X-ray Diffraction

Residual stresses were measured with a Stresstech Xstess 3000 G3R^®^ apparatus (Stresstech Oy, Vaajakoski, Finland), to evaluate the residual stresses. These tests were performed using the Kα-Chromium radiation coupled with a Vanadium filter and following the ASTM E2860-12 standard [35]. The measurements were performed according to the SAE International standard HS-784/2003 Residual Stress Measurement by XRD [36].

A depth profile of the residual stresses was drawn using electropolishing, following the ASTM E1558-14 standard [37]. This operation was performed by means of an electro-polishing facility, removing step by step the thin layers of material, using acetic acid (CH_3_COOH) as an electrolyte. The results of the in-depth residual stress measures were corrected using the method described by Moore and Evans [38]. The thickness that was removed was checked with a micrometer (Mitutoyo Italiana S.r.l., Lainate, Italy).

### 2.6. Vickers Microhardness

Vickers microhardness tests were performed following the ASTM E92-03 standard [39]. The hardness of the different phases was measured using a 100 gf load with a loading time of 15 s. On the other hand, the surface hardness was measured through a 200 gf load with an indentation time of 15 s.

### 2.7. Scanning Electron Microscope Analysis

The local chemical composition of the two constituent phases of the samples was acquired through a scanning electron microscope (SEM) Zeiss EVO 50^®^ (Carl Zeiss Microscopy GmbH, Jena, Germany, using dispersive X-ray microanalysis (SEM-EDS, Carl Zeiss Microscopy GmbH, Jena, Germany) and secondary electron detector (SEM-SE, Carl Zeiss Microscopy GmbH, Jena, Germany).

This analysis examines the distribution of each alloying element between the ferritic and austenitic phase, which is crucial to optimize the mechanical and corrosion resistance properties [23,24].

### 2.8. Roughness

Roughness measurements were performed on the surface of the shot peened samples, using a stylus Mahr PGK MFK-250^®^ tester (Mahr GmbH, Göttingen, Germany) with a tip radius of 2 μm with a vertical measure range of ±250 μm. The data were collected following the UNI EN ISO 4288-2000 standard [40]. The translational speed during the measurement was set at 0.5 mm/s along an exploration length of 5.6 mm. The base length was 0.8 mm and the total evaluated length was 4.0 mm. Data were filtered using a Gaussian filter function with a wavelength cutter of 0.8 mm. From the surface profiles, obtained with the optical microscopy, it is possible to evaluate the surface roughness through the fractal dimension of the surfaces along 1 mm of the surface profile. The images were processed by Image Pro Plus^®^ software (version 4.5, Media Cybernetics Inc., Rockville, MD, USA), using a ruler-counting method evaluation of fractal dimension.

### 2.9. Salt Spray Fog Tests

The corrosion resistance of the specimens was evaluated via salt spray fog tests, which were executed for 168 h following the ASTM B117-16 standard [41]. The chamber temperature was set at 35 ± 2 °C and the sprayed solution was prepared by dissolving 50 g/L of NaCl in distilled water. The solution pH was controlled to be between 6.5 and 7.2 and its flux, feeding the chamber, was equal to 1–2 mL/h. Afterwards the corrosion resistance properties of the samples were evaluated by controlling the mass variation as prescribed by UNI EN ISO 9227-12 [42]. The measuring instrument is featured by an error equal to 0.1 mg.

## 3. Theory and Calculation

### 3.1. Shot Peening Coverage

In recent years, models have been developed to theoretically predict and control the coverage percentage of the shot peening processes. The most famous model was developed by Kirk and Abyaneh. This model, supported by theoretical and practical evidence, asserts that, as the amount of peening increases, the coverage points to an exponential approach that reaches 100%. According to this evidence, Avrami’s simplest equation (Equation (1)) is employed to relate the coverage to the amount of peening, expressed as [43]:

(1) $$C_{t}\%\;=100*\left[1-e^{-A*t}\right]$$

where $C_{t}\%$ is the coverage after a time t and A corresponds to the indent rate. This parameter is also given by (Equation (2)) [43]:

*A* = −*ln*[(100 − *C*_*t*0_%)/100] (2)

where $C_{t0}\%$ is the coverage percentage measured after a shot peening process during *t*_0_. Through the coverage percentage measured after a fixed time, it is possible to calculate the indent rate A and, then, the time t, needed to reach the desired coverage.

The SAE J2277 standard [44] sets a target for coverage, defined as “full coverage”, being equivalent to 98% actual coverage [43,45].

### 3.2. Austenite Volume Fraction Measurements via X-ray Diffraction

Measurements of austenite content through XRD are based on the calculation of the diffraction peak areas of both the ferritic and austenitic phase, since these area are proportional to the volumetric content of each phase. Thus, the austenite content can be calculated using the equation (Equation (3)) [46]:

(3) $$\;V_{\gamma }=\left(1-V_{c}\right)*\left(\frac{1}{q}*\overset{q}{\underset{j=1}{\sum }}\frac{I_{\gamma j}}{R_{\gamma j}}\right)/\left[\left(\frac{1}{p}*\overset{p}{\underset{j=1}{\sum }}\frac{I_{\alpha i}}{R_{\alpha i}}\right)+\left(\frac{1}{q}*\overset{q}{\underset{j=1}{\sum }}\frac{I_{\gamma j}}{R_{\gamma j}}\right)\right]$$

where $V_{\gamma }$ is the austenite content, $V_{C}$ is the carbides content, q is the number of austenitic peaks (hkl), $I_{\gamma j}$ is the area of the j−peak of austenite and *R_γj_* is a theoretic parameter of the area under the intensity graph for the austenitic phase, which depends on the interplanar spacing (hkl), the Bragg angle, the crystalline structure, and the composition of the measured phases. In parallel, p is the number of ferritic peaks (hkl), *I_αi_* is the area of the i−peak of ferrite, and $R_{\alpha i}$ is a theoretic parameter of the area under the intensity graph for the ferritic phase, which depends on the interplanar spacing (hkl), the Bragg angle, the crystalline structure and the composition of the measured phases.

To obtain reliable results it is crucial that the diffraction peaks fit. In this work, the evaluation of the diffraction peaks of the austenitic phase at 130 and 80 degrees was carried out by using a parabolic function to reduce the background noise and the Gauss-function to fit the curve. For the ferrite phase diffraction the PearsonVII-function was used to fit the peaks at 156.4 and 106.1 degrees, and a linear function was used for noise reduction [46,47].

Table 3 shows the parameters used in the austenite measurements. As XRD intensity is influenced by the presence of preferred orientations, the effect of textures should be corrected to estimate more precisely the volume fractions of the phases. However, it would not affect the comparison between the data collected before and the after shot peening process. Since the comparison among the different samples is treated mainly qualitatively and the crystallographic texture effect on XRD austenite content measurement would affect only the cold drawn “CD” samples, it may be neglected.

### 3.3. Residual Stresses Measurements via X-ray Diffraction

Residual stresses were measured via XRD with Kα-Chromium radiation (2θ = 156.1°, 30 kV, 6.7 mA) for α-phase (211) and with Kα-Chromium radiation (2θ = 128.8°, 30 kV, 6.7 mA) for γ-phase (220). The stresses were determined by the sin^2^ Ψ method, measuring 11 angles, and 1 mm^2^ of the irradiated area. For the ferrite diffraction peak at 156.1°, a pseudo-Voigt-function was employed to fit the peak and a parabolic function for noise reduction. The diffraction peak of the austenitic phase at 128.8° degrees was carried out by using a parabolic function to reduce the background noise and the Gauss-function to fit the curve. The average penetration depth of Kα-Chromium radiation is about 5 μm for ferrite and 10 µm for austenite [49,50,51,52,53].

### 3.4. Alloying Elements Partitioning

As previously mentioned, the properties of super duplex stainless steels are optimized whenever a proper control of the chemical composition is reached. This means not only a control on the overall composition, but also a control in the partitioning of the alloying elements between the two main constituent phases. To evaluate the distribution of the alloying elements a partition coefficient $P_{\gamma /\alpha }^{X}$ has been used. This partition coefficient is given by Equation (4):

(4) $$P_{\gamma /\alpha }^{X}=\left[X_{\gamma }]/[X_{\alpha }\right].$$

where $X_{\gamma }$ is the concentration of the alloying element X (expressed as wt %) within the austenitic phase and, on the other hand, *X_α_* is the concentration of the alloying element X (expressed as wt %) within the ferrite [23].

### 3.5. Surface Roughness Fractal Analysis

Using the surface profiles obtained through optical microscopy, it is possible to evaluate the surface roughness through the fractal dimension of the surfaces. The original images of the cross-section (5 images of a width corresponding to approximately 1 mm of the surface profile) are processed by Image Pro Plus^®^ software (version 4.5, Media Cybernetics Inc., Rockville, MD, USA). This software applies a ruler-counting method to the evaluation of the fractal dimension for the surface profiles.

The surface profile is covered with rulers of length d. If the profile is completely covered with N rulers, the fractal geometry shows the following relationship Equation (5):

(5) $$N\left(d\right)=\mu d^{-D}$$

where D is the fractal dimension and *μ* is a positive constant [54,55].

### 3.6. Full Width at Half Maximum (FWHM)

XRD techniques are able to detect and separate the contribution of microscopic stresses, or microstresses, within the crystals. Microscopic stresses result from imperfections in the crystal lattice and are without direction. Thus, microstresses vary from point to point within the crystal lattice, broadening the diffraction peak, due to the alterations in the lattice spacing. Then, microstresses can be determined from the diffraction peak breadth. In this work, the width of the diffraction peak has been calculated from the width of the function fitted to the diffraction-peak profile during macrostress measurement. Then, it has been quantified precisely as the width at half the height of the diffraction peak (Full Width at Half Maximum, FWHM). This quantity is assumed as an index of the hardening of the material [56,57].

## 4. Results

An investigation of the behavior of AISI F55 (UNS S32760) super duplex stainless steel, dealing with a mechanical surface treatment, such as shot peening, has been performed. The obtained data identifies how microstructural features affect corrosion behavior and are affected by this process.

### 4.1. Optical Microscopy

**Figure 1:**
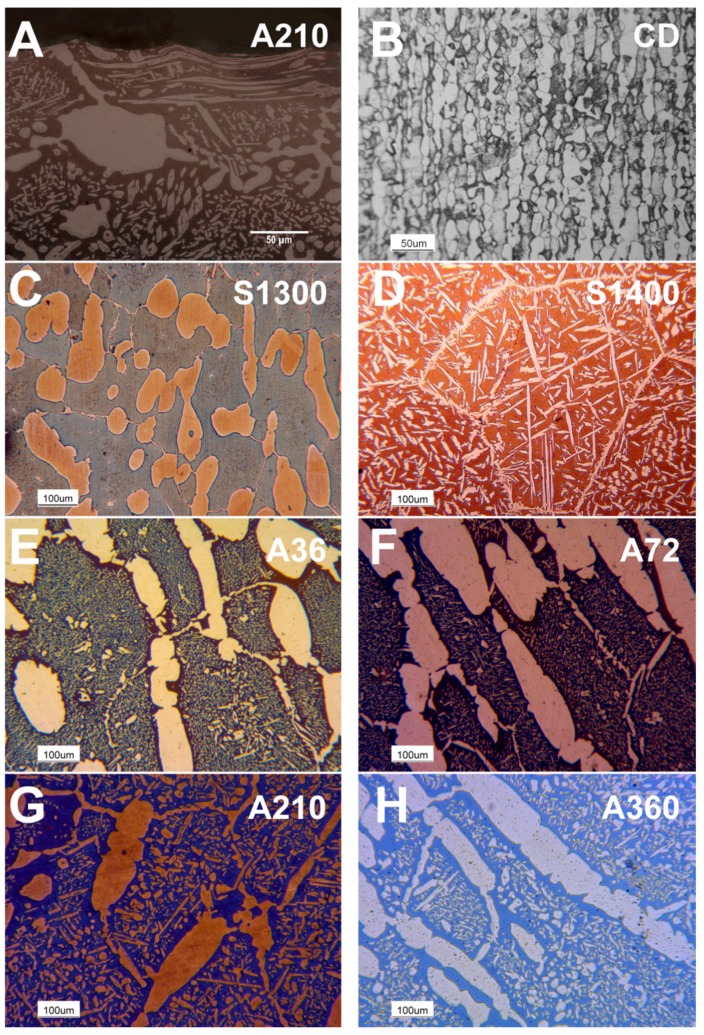
Sample microstructure through optical microscopy after Beraha’s tint etch: Surface section of the sample annealed at 1080 °C for 210 s/mm (**A**); Core section of the cold drawn reference specimen (**B**); The solution annealed at 1300 °C (**C**); The solution annealed at 1400 °C (**D**) and the samples annealed at 1080 °C for 36 s/mm (**E**); 72 s/mm (**F**); 210 s/mm (**G**) and 360 s/mm (**H**).

Optical microscopy reveals the complexity of the microstructure featuring this steel grade. The thermal treatments drastically modify the microstructure. Cold drawn samples present the typical “pancake” microstructure (Figure 1B). S1400 samples show the austenitic phase contouring the ferritic grains as allotriomorphs and within the grains as Widmanstätten γ-austenite plates (Figure 1D). S1300 samples present a microstructure characterized by roundish austenitic grains dispersed within the ferritic matrix (Figure 1C). The annealing treatment triggers the secondary austenite precipitation (Figure 1E), followed by the growth of the precipitates with ongoing thermal treatment (Figure 1F). The static recovery of the ferritic grains is reported to occur between 210 s/mm (Figure 1G) and 360 s/mm (Figure 1H) of the holding time. Moreover, the effect of the deformation by the performed shot peening surface treatment can be detected by observing the distortion of the sub-surface austenitic grains (Figure 1A).

### 4.2. Austenite Volume Fraction Measurements via X-ray Diffraction

The austenite content of the samples measured via XRD in the same area before and after the shot peening process is reported in Table 4, where TTγ% is the austenite amount (%) after the thermal treatment. The data evinces a strong decrease of the austenitic phase volume fraction after the shot peening process, revealing the capacity of this treatment to induce phase changes in the steel microstructure.

### 4.3. Residual Stresses Measurements via X-ray Diffraction

**Figure 2:**
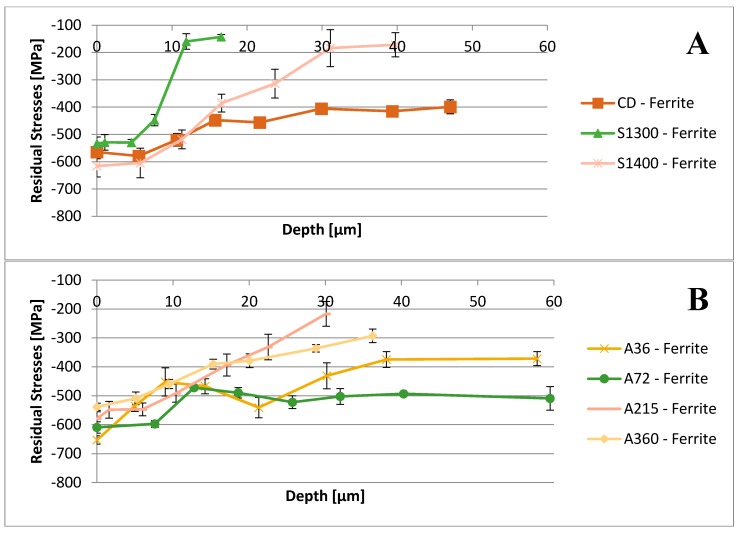
Depth profiles of the residual stresses induced by shot peening treatment within the ferritic phase: CD, S1300, S1400 samples (**A**) and A36, A72, A215, A360 (**B**).

**Figure 3:**
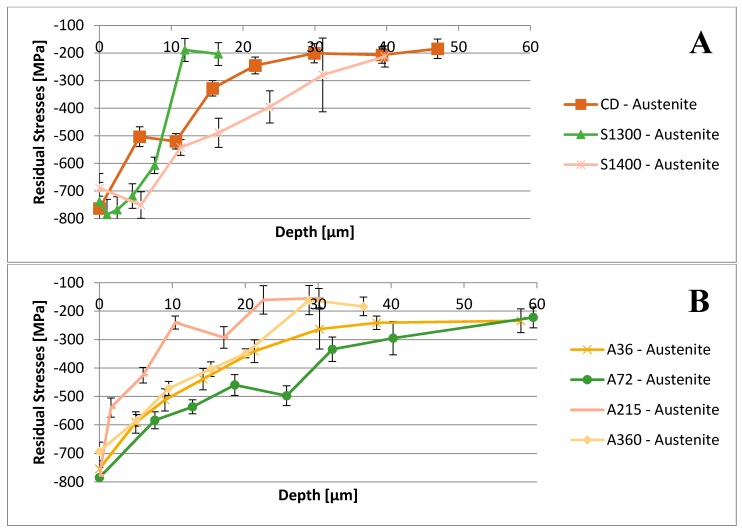
Depth profiles of the residual stresses induced by shot peening treatment within the austenitic phase: CD, S1300, S1400 samples (**A**) and A36, A72, A215, A360 (**B**).

The depth profiles of residual stresses induced by the shot peening treatment have been recorded and are reported in Figure 2 and Figure 3.

### 4.4. Vickers Microhardness

**Figure 4:**
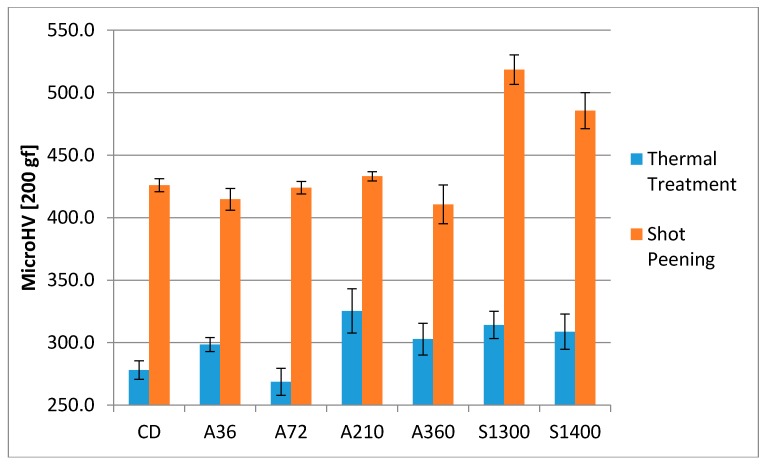
Surface hardness before and after shot peening treatment.

Another beneficial effect of shot peening is the increase in surface hardness, which is responsible for the better behavior of the processed product in some working conditions, such as fretting or erosion. The increase in surface hardness achieved through the shot peening process was measured via Vickers microhardness tests and the results are reported in Figure 4. All the samples show a strong increase in surface hardness, more than 100 HV. The highest increase was found in the solubilized samples, around 200 HV, giving the surface hardness near 500 HV.

**Figure 5:**
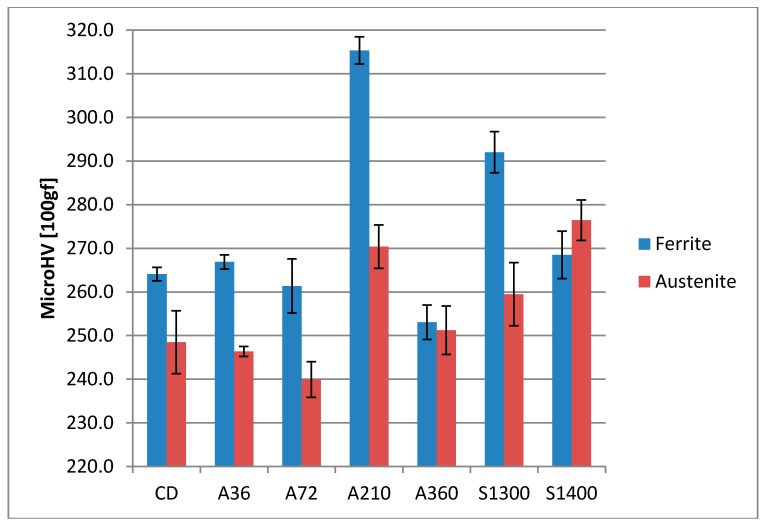
Vickers microhardness of the two constituent phases.

Furthermore, through the Vickers microhardness tests, the hardness of the two main constituent phases have been evaluated. The results are reported in Figure 5. The ferritic phase presents a higher hardness with respect to the austenite phase in every specimen, except in the solubilized at 1400 °C samples. In the latter case, the two phases display similar Vickers microhardness values. This uniformity in the mechanical properties can be related to the similar chemical composition of the ferritic and austenitic phases and, consequently, to solid solution hardening.

### 4.5. Scanning Electron Microscope Analysis

**Figure 6:**
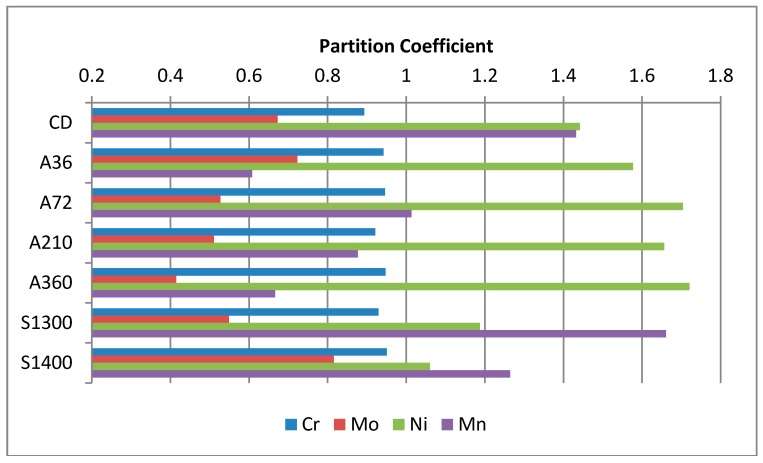
Alloying element partitioning between the austenite and ferrite as stated in Equation (4).

Table 5 shows the local chemical composition of the two constituent phases, ferrite and austenite, obtained by SEM-EDS analysis. Each alloying element preferentially redistributes in one of the phases. Their behavior is extremely important to optimize the final properties of this steel grade, both in terms of the mechanical and corrosion resistance properties. For instance, strong mis-balances of the alloying elements may trigger galvanic corrosion micro-couples. Thus, a correct partitioning should be achieved. In Figure 6, the partitioning coefficient of the main alloying element, calculated as reported in Equation (4), has been plotted. It can be observed that samples solubilized at 1400 °C result in the most mixed composition, which feature partitioning coefficients closer to the unity value than other specimens.

### 4.6. Roughness

Since shot peening is a mechanical surface process, it modifies the surface morphology. Thus, roughness parameters after the shot peening of the samples were measured by means of a stylus Mahr PGK MFK-250^®^ tester (Mahr GmbH, Göttingen, Germany). In Table 6 the resulting data are presented, following the UNI EN ISO 4287-1996 standard [58]. It is interesting to underline the absence of any relationship, which could be conjectured, between the surface hardness of the samples and their final roughness. Further, it must be highlighted that the smoothest surface after shot peening is obtained in solubilized at 1400 °C samples. The same results are given by the fractal dimensions data, which can include hook-shaped indentations, evincing the surface damages induced by the shot peening process.

### 4.7. Salt Spray Fog Tests

Finally, the effect of the shot peening treatment on the pitting corrosion resistance properties of the steel were checked through a salt spray fog test. The results and their projection after one year of exposure are given in Table 7. The not univocal effect of the shot peening process on the pitting corrosion resistance properties of the investigated steel should be highlighted. All the samples display a good pitting corrosion resistance after the thermal treatment, closer to the behavior of the commercial cold drawn reference. In detail, the cold drawn reference and the thermally annealed A36 samples increased their weight after the salt spray fog test, which is related to oxidation phenomena. Almost all the specimens show a worsening of their corrosion resistance after the shot peening treatment. On the other hand, the weight of the solubilized S1400 and the annealed A360 samples do not vary after the corrosion tests. Furthermore, after a visual inspection of the surface by optical microscopy, corrosion products have not been detected on the solubilized at 1400 °C sample, proving its consistent increase in pitting corrosion resistance.

## 5. Discussion

The optical microscopy analysis (Figure 1) shows the typical complex microstructure of AISI F55-UNS S32760 super duplex stainless steel, including the secondary austenite precipitates present in their morphologies: martensitic-shear precipitation and Widmanstätten structures [26]. The distortion of the sub-surface austenitic grains demonstrates the deformation induced by the shot peening, which is a purely mechanical surface treatment. The presence of any other phase different from the former ones (austenite and ferrite), especially the most detrimental ones in this kind of steel (σ and χ) [23,24,25], have not been detected by optical microscopy, neither before or after the shot peening treatment [59].

However, the austenitic content after shot peening treatments, measured via XRD analysis, reveals a large decrease of the austenite content after the shot peening treatment (Table 4). Thus, the occurrence of a stress/strain-induced microstructural modification has been identified. The martensitic phase can be generated from the austenite by deformation through two mechanisms. At room temperature, the martensitic transformation is ruled by stain-induced mechanisms [59,60]. Although it has a low stacking fault energy, austenite in duplex stainless steel is usually not prone to strain-induced martensite formation, since the duplex microstructure modifies the mechanisms of deformation. On the other hand, the decrease in the austenite volume fraction, measured by XRD but not detected by optical microscopy, coupled with the absence of any other phase nucleation, proves the generation of strain-induced martensite through the shot peening treatment [59,60,61].

This strain-induced martensite enhances the austenite work hardening and usually affects the corrosion resistance, since it increases the number of active anodic sites at the surface [60,61].

Further, in-depth profiles of the residual stresses measured by XRD have been drawn for each specimen (Figure 2 and Figure 3). All the samples show the presence of compressive residual stresses at the surface and in a thin layer under the surface, due to the shot peening treatment.

Residual stresses measured in the inner areas of the samples in the ferritic phase testify also to the presence of the residual stresses given by the previous thermo-mechanical history of the specimens. In detail, the residual stresses of the CD sample are related to the cold-forming processes, featuring this specimen. On the other hand, the residual stresses in the core of the annealed A36, A72, A210, and A360 samples are generated by the secondary austenite precipitation process, which occurred during the thermal treatment. Indeed, this precipitation generates compressive stresses within the ferritic matrix, due to the military character of the martensitic-shear and Widmanstätten precipitation processes. Further, with the ongoing of the annealing treatment, the relaxation of these residual stresses is observed. On the other hand, thermally generated residual stresses have not been detected within austenite, since its low stacking fault energy promotes recrystallization processes and the consequent annihilation of these stresses [62,63,64].

As a result, the samples, which are mainly affected by the shot peening treatment, are solubilized at 1300 °C and at 1400 °C. Indeed, they experience a larger increase in the compressive residual stresses at the surface in the ferritic phase. Consequently, this results in a larger enhancement of the surface hardness in comparison to the other investigated conditions. This consideration should be extrapolated without the contribution of the austenitic phase, since it would result in marginal change, which is expected by micromechanics [65,66,67]. As expected, this trend is observed in the increase in surface hardness (Figure 4).

Furthermore, these results, which display high surface compressive residual stresses even after an annealing thermal treatment, are related to two main reasons. The first is the thermal path of the treatment. The very high temperature of the treatment followed by the water quench achieves very high cooling rates, generating residual stresses in the material. The second reason is related to the high temperature phase transformations of AISI F55-UNS S32760 Super Duplex Stainless Steel displacive precipitations, the recrystallizations that not occur at the same time for the 2 main constituent phases, and the different stress-strain partitioning that occurs between the two phases. [21,25,26,65,66,67]. In addition to the data presented in this work, two other sets of XRD measurements were made on samples prepared following exactly the same experimental and thermal paths: the first at around 200–300 μm depth and the other one much deeper on the samples sections. These two sets of measures have been made with two different XRD devices, one at Universidad de Oviedo and the other at Politecnico di Milano. Both of the obtained results are in accordance with the data presented in this work. These two sets of results identify the depth measure limits.

Focusing on the penetration depth of the shot peening treatment, it can be observed that this surface mechanical treatment affects a thin superficial layer. In detail, this layer is around 20–30 μm deep, and even less in the sample annealed at 1300 °C (10–12 μm deep).

Regarding the shape of the residual stresses depth profiles, further considerations should be highlighted. The compressive residual stress state induced by the shot peening can be described by two overlaid phenomena. First, Hertzian pressure generates compressive residual stresses with a maximum below the surface. Second, the surface layer undergoes plastic stretching, leading to residual stresses with the maximum at the surface itself. The predominant effect depends upon the strength of the peened material. The substrate material and its microstructure may also contribute to the compressive residual stresses depth profile through a third process: stress/strain induced martensite transformation. The constraint, given by the volume dilation and exerted by the surrounding material, leads to a further deepening of the compressive residual stress values, moving the compressive residual stresses peak within the subsurface region, where the maximum Hertzian pressure is reached [48,65,68,69].

The compression residual stresses peaks occur in the subsurface region, due to the initial surface hardness of the samples (Figure 4) at the threshold values of 300–350 HV. The specimens solubilized at 1300 °C and at 1400 °C follow this behavior for both the constituent phases. On the other hand, A210 sample, which has the higher surface hardness, does not display the same behavior. In detail, its austenitic phase clearly shows the maximum of its compressive residual stresses profile located at the surface. This behavior is demonstrated by the very little quantity of strain induced martensitic transformation occurring in this sample, which results not sufficient to shift the maximum of the compressive residual stresses to the subsurface region.

Focusing on the corrosion resistance properties, the experimental results show an evident difference among the tested samples (Table 7). First, the significant chromium, nickel, and molybdenum content of this alloy (Table 5) must be underlined, resulting in a strong capability to counteract external degradation attacks, which is typical of the investigated steel grade. Assuming this, in the test environment the surficial degradation of the metallic alloy is caused by three mechanisms: generalized corrosion, pitting corrosion, and galvanic corrosion between the two constituent phases. The collected data describe the overall phenomenon [70,71].

Before the shot peening treatment, specimens show a very low corrosion rate and, consequently, a very high pitting corrosion resistance. A slight increment in weight after the salt spray fog test has been recorded by the samples CD and A36. This behavior can be linked to generalized corrosion and the development and the thickening of the surface passive film on the surface. Thus, the pitting phenomena on these samples has not been recorded and has not been identified by visual inspection via optical microscopy. On the other hand, this visual inspection has detected the presence of corrosion products. Then, the projection at one year of exposure for these samples is not meaningful, since over such a long time-interval the pitting corrosion is expected to be trigged.

In general, after the shot peening treatment, the specimens become damaged by the chloride-rich environment, showing a lower pitting corrosion resistance than the thermally treated samples. This can be related to the surface defects generated by the surface mechanical treatment, which would act as initiation sites for the pitting phenomena. The presence of strain-induced martensite increases the number of active anodic sites at the surface. Further, the cold-worked surface layer shifts the corrosion potential of the austenite, which is the nobler phase to the anodic direction, decreasing the overall corrosion resistance of the alloy [17].

On the other hand, the samples S1400 and A360 are not affected by the degradation phenomena in the gravimetric tests. Also, the visual inspection via optical microscopy does not record the presence of any pitting phenomena on these samples. However, corrosion products have been detected on the surface of the sample annealed for 360 s/mm and shot peened. Again, the projection at one year of exposure for these samples is not meaningful, since over such a long time-interval the pitting corrosion is expected to occur [19]. Then, the data assigned to the solubilized at 1400 °C and shot peened sample display the best behavior in a chloride-rich environment, appearing in contrast with those collected for all the other shot peened samples. This result can be explained considering different aspects.

**Figure 7:**
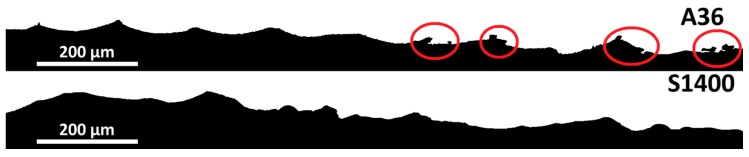
Surface sections of the shot peened samples previously annealed at 1080 °C for 36 s/mm (**above**) and solubilized at 1400 °C for 72 s/mm (**below**): cracks and hook-shaped defects, which act as pitting corrosion initiation sites, have been highlighted.

First, the solubilized at 1400 °C sample achieved quite a pristine surface after the mechanical surface treatment, as observed by the fractal analysis (Table 6). Indeed, in this case the absence of cracks and hook-shaped defects prevents the formation of narrow crevices, which actuate as pitting corrosion initiation sites (Figure 7) [72,73].

Further, the compressive residual stresses induced by the shot peening increase the duplex stainless steels pitting corrosion resistance. These three effects may be responsible for the increased corrosion resistance of the samples. First, compressive residual stresses in the surface layer have a beneficial contribution to the generation and growth kinetics of the passive film, improving the electrochemical corrosion resistance. Further, with the increasing compressive stresses, the corrosion potential becomes nobler because the stress field has been proven to enhance the Cr/Fe ratio in the passive film [17]. Finally, compressive residual stresses lead to the reduction of the derogatory effects of inclusions on the treated surface [59] and prevent pit initiation on the inclusions at low potential without changing the pitting potential [59], enhancing the corrosion resistance [74,75,76,77,78].

However, these features do not completely explain the good results obtained by the specimen solubilized at 1400 °C also under the mechanical point of view. Micromechanics highlight different aspects, which could have significant results for this aim. First, the austenitic phase is the most attracted by a mechanical treatment. Second, homogeneity at the surface plays a key role for both the mechanical and corrosion resistance properties [23]. Thus, to obtain it after the shot peening process, the initial microstructure must be as uniform as possible, by focusing mainly on austenite as previously stated [79,80]. Taking in account all these aspects, the sample solubilized at 1400 °C displays the most suitable initial microstructure. Indeed, it appears as the most homogeneous in terms of austenite morphology and distribution (Figure 1), the mechanical properties of the two constituent phases (Figure 5), and also the chemical element distribution between the two phases (Table 5 and Figure 6).

**Figure 8:**
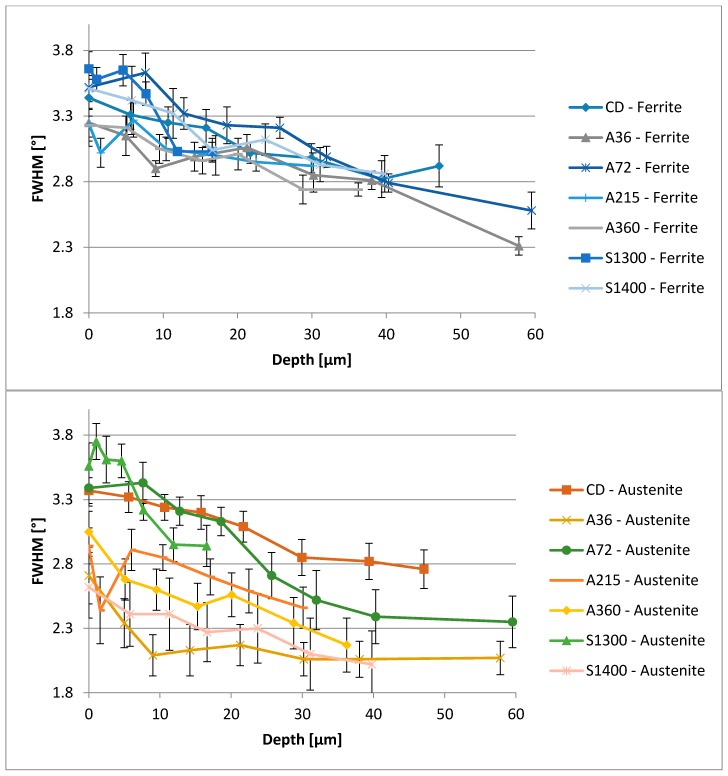
Full Width at Half Maximum (FWHM) parameters obtained via XRD spectra, indicating the presence of crystal lattice imperfections within ferrite (**Above**) and austenite (**Below**).

Further, this specimen is also the most homogeneous in the energy dissipation modes, featuring its microstructure and displaying impact energy storage, provided by the shot peening process. Indeed, the materials continuum may store the energy conferred by the blasting medium impact, generating imperfections within the crystal lattice. These imperfections result in microscopic stresses and can be measured through the Full Width at Half Maximum (FWHM) of the XRD spectra. The collected data are grouped in Figure 8. Microscopic stresses are without direction, but, since they are generated by a directional process such as shot peening, their presence should be limited to achieve the maximum homogeneity within the microstructure. The depth profiles of the FWHM values reflect the trend of the residual stresses, to which are linked [56,57]. Microstresses measured at the surface within the ferritic phase are quite similar for all the samples with narrow data scattering. Further, since ferrite is less prone to deformations, these microstresses are less significant [65,66,67]. On the other hand, results from the austenitic phase of the surface samples show a broadening, which increases the significance of the data. The solubilized at 1400 °C sample results are less affected by this phenomenon, demonstrating the tendency of this microstructure to accommodate energy in a different manner.

A further method used by the microstructure to accommodate strains and to store the energy given by the impact of the blasting medium is the generation of staking faults. For face centered cubic (f.c.c.) lattices, such as austenite, one of these mechanisms is the generation of twins. Twins are 2-dimensional defects; they intrinsically create anisotropy in the surrounding microstructure [81]. Thus, to achieve a homogeneous microstructure their presence should be limited.

However, twins may be thermally generated; thus, benchmark values for each sample should be measured to assess the increase, due to the shot peening process [82]. The number of twins per area has been counted, using oxalic acid electrochemically etched micrographs, in both regions: near the surface in the mechanically affected layer and in the core of the samples. In Table 8, the collected data are shown. The increase in twins concentration at the samples surface evinces their “mechanical” nucleation, which is related to the storage of the impact energy of the blasting medium.

The completely different behavior of the sample solubilized at 1400 °C can also be highlighted. This is due to the austenitic grain morphology of these specimens. Indeed, it is composed only by allotriomorph austenite and Widmanstätten austenite plates. In the first morphology, twins cannot be generated because of the presence of grain boundaries, which are too close. In the latter case, twins cannot be generated since the Widmanstätten austenite plates are generated by the surrounding defects and a set of periodic dislocations share the same direction, nearly parallel to the close-packed directions [83,84]. This behavior ensures a more uniform and homogeneous microstructure with respect to the other samples, in which these 2-dimensional defects intrinsically create anisotropy.

Finally, the last possible energy dissipation mode featuring these microstructures is the strain induced martensitic transformation. Since it is a 3-dimensional process, it intrinsically results in the most uniform and homogeneous outcome. As reported in Table 4, the specimens solubilized at 1400 °C widely exploit this energy dissipation phenomenon to store the impact energy provided by the shot peening process. Thus, its microstructure results are the most homogeneous, considering this aspect.

## 6. Conclusions

Specimens in F55-UNS S32760 super duplex stainless steel have undergone different preparatory thermal treatments in order to investigate the effect of an annealing thermal treatment or a higher temperature solution thermal treatment, on the final properties of the shot peened samples. The working parameters of the shot peening treatments were defined by previous studies, to obtain the higher fatigue resistance.

All the shot peened specimens obtained the generation of the surface compressive residual stresses and a surface hardness increase. Further, the 1400 °C solution thermal treatment results in the best initial condition before the shot peening treatment for corrosion resistance. This can be pointed out as a result of several features of the combination of improvements:

- The higher beneficial effect developed by the shot peening process, in terms of compressive residual stresses;
- The depth and the profile’s shape and the mechanically affected layer;
- The surface hardness increase;
- The surface roughness decrease;
- The higher pitting corrosion resistance.

These results can be explained via micromechanics assumptions, which would define as the best initial condition as the most homogeneous one, in terms of the microstructure. The 1400 °C solution thermal treatment ensures the achievement of this condition, considering many aspects:

- The microstructure morphology;
- The mechanical properties of the two composing phases;
- The chemical elements distribution within the phases;
- The energy dissipation modes exploited by the microstructure.

## Figures and Tables

**Table 1:** Summary of the performed thermal treatments.

Sample	Thermal Treatment
CD	Cold Drawn
A36	1300 °C, 60 s/mm + water quenched + 1080 °C, 36 s/mm + water quenched
A72	1300 °C, 60 s/mm + water quenched + 1080 °C, 72 s/mm + water quenched
A210	1300 °C, 60 s/mm + water quenched + 1080 °C, 210 s/mm + water quenched
A360	1300 °C, 60 s/mm + water quenched + 1080 °C, 360 s/mm + water quenched
S1300	1300 °C, 72 s/mm + water quenched
S1400	1400 °C, 72 s/mm + water quenched

**Table 2:** Avrami equation parameters.

Sample	Average Coverage after 1 s of SP	Coverage Standard Deviation after 1 s of SP	AR Coeff.	*t* (98%) [s]
CD	65	3.0	1.05	3.71
A36	89	3.7	2.18	1.79
A72	67	2.9	1.10	3.57
A210	83	3.9	1.75	2.24
A360	77	4.1	1.45	2.69
S1300	60	3.3	0.92	4.28
S1400	73	2.6	1.31	2.98

**Table 3:** Parameters used in the X-ray diffraction (XRD) measurements [48].

Phase	(hkl)	2θ [°]	R	Poisson’s Coefficient	Young’s Modulus [MPa]
Ferrite	200	106.1	18.92	0.28	220,264
	211	156.4	183.13		
Austenite	200	80	26.42	0.28	207,039
	220	130	51.54		

**Table 4:** Austenitic phase volume fraction measured via XRD technique in the same areas of the specimens, before and after shot peening process.

Sample	Thermal Treatment		Shot Peening		Δ	Δ/(TTγ%)
	% Austenite	% Uncertainties	% Austenite	% Uncertainties	% Austenite	Ratio
	[Vol %]	[Vol %]	[Vol %]	[Vol %]	[Vol %]	
CD	55.3	2.3	38.1	1.7	−17.2 ± 4.0	−0.31 ± 0.03
A36	54.6	1.8	47	3.9	−7.6 ± 5.7	−0.14 ± 0.02
A72	47.7	3.4	33	3.7	−14.7 ± 7.1	−0.31 ± 0.06
A210	45.9	2.5	41	1.3	−4.9 ± 3.8	−0.11 ± 0.01
A360	53.7	2.7	23.5	2.2	−30.2 ± 4.9	−0.56 ± 0.08
S1300	31.8	2.5	25.6	1.4	−6.2 ± 3.9	−0.19 ± 0.03
S1400	37.3	1.7	25.3	2.1	−12 ± 3.8	−0.32 ± 0.04

**Table 5:** Chemical composition measured within austenitic and ferritic phases.

Sample	Phase	% Fe	% Cr	% Ni	% Mo	% W	% Cu	% Mn
CD	γ	62.14	24.13	8.52	3.19	0.61	0.78	0.63
	α	60.46	27.01	5.91	4.74	0.89	0.55	0.44
A36	γ	61.51	25.05	8.06	3.76	0.86	0.33	0.45
	α	60.53	26.59	5.11	5.2	1.39	0.44	0.74
A72	γ	60.10	22.87	9.05	4.28	1.82	1.11	0.77
	α	58.50	24.18	5.31	8.12	2.06	1.07	0.76
A210	γ	61.15	24.5	7.95	3.65	1.48	0.71	0.57
	α	58.61	26.6	4.80	7.15	1.65	0.54	0.65
A355	γ	61.75	24.76	7.71	3.52	1.42	0.53	0.32
	α	57.37	26.13	4.48	8.49	1.69	1.36	0.48
S1300	γ	61.22	24.21	7.85	3.03	1.36	1.36	0.98
	α	59.17	26.04	6.61	5.52	1.60	0.47	0.59
S1400	γ	61.19	24.38	7.41	3.89	1.44	0.81	0.88
	α	59.56	25.64	6.99	4.77	1.53	0.82	0.70

**Table 6:** Surface roughness test results.

Average Roughness	Ra	Rq	Rz	Rmax	Rp	Rv	Fractal Dimension
	[μm]	[μm]	[μm]	[μm]	[μm]	[μm]	
CD	3.5 ± 0.2	4.6 ± 0.21	22.9 ± 1.7	27.6 ± 4.6	13.1 ± 1.4	9.7 ± 1.0	1.018 ± 0.0010
A36	3.7 ± 0.12	4.5 ± 0.16	19.7 ± 1.9	23.7 ± 2.8	11.9 ± 1.9	7.7 ± 0.4	1.023 ± 0.0034
A72	3.8 ± 0.13	4.7 ± 0.16	21.3 ± 1.2	26.7 ± 3.7	12.7 ± 1.1	8.5 ± 0.7	1.015 ± 0.0010
A210	3.7 ± 0.26	4.6 ± 0.27	20.4 ± 2.5	24.6 ± 2.5	12.4 ± 1.3	8.0 ± 0.8	1.020 ± 0.0011
A360	3.9 ± 0.13	4.6 ± 0.34	21.1 ± 0.8	27.5 ± 1.7	11.9 ± 0.6	9.3 ± 1.0	1.018 ± 0.0030
S1300	3.7 ± 0.13	4.6 ± 0.12	20.7 ± 1.3	26.5 ± 3.9	12.5 ± 1.0	8.2 ± 1.0	1.018 ± 0.0021
S1400	3.5 ± 0.15	4.3 ± 0.16	20.6 ± 0.7	24.2 ± 1.5	11.3 ± 0.5	9.0 ± 0.3	1.013 ± 0.0018

**Table 7:** Results of the salt spray fog tests.

Sample	Thermal Treated—Δ Weight		Shot Peened—Δ Weight	
	1 Week Exposure [g/(mm^2^ × Week)]	1 Year Projection [g/(mm^2^ × Week)]	1 Week Exposure [g/(mm^2^ × Week)]	1 Year Projection [g/(mm^2^ × Week)]
CD	0.10	--	−1.12	−58.65
A36	0.07	--	−0.52	−27.06
A72	−0.23	−11.85	−0.75	−38.96
A210	−0.15	−7.66	−0.59	−30.66
A360	−0.11	−5.68	0.00	--
S1300	−0.10	−5.13	−0.92	−47.86
S1400	−0.13	−6.74	0.00	--

**Table 8:** Number of twins per area.

Sample	Core	Surface	Δ
	[n° Twins/mm^2^]	[n° Twins/mm^2^]	[n° Twins/mm^2^]
CD	1379	3013	1633
A36	143	416	273
A72	70	102	32
A210	40	126	86
A360	165	488	323
S1300	701	1431	729
S1400	0	0	0
